# Serum cystatin C levels relate to no-reflow phenomenon in percutaneous coronary interventions in ST-segment elevation myocardial infarction

**DOI:** 10.1371/journal.pone.0220654

**Published:** 2019-08-01

**Authors:** Chao Cheng, Xiao-Bo Liu, Shao-Jie Bi, Qing-Hua Lu, Juan Zhang

**Affiliations:** 1 Department of Cardiology, The Second Hospital of Shandong University, Jinan, Shandong Province, China; 2 Shandong Blood Center, Jinan, Shandong Province, China; Erasmus Medical Center, NETHERLANDS

## Abstract

**Background/Aim:**

No-reflow is a serious and frequent event during primary percutaneous coronary intervention (PPCI) for acute ST segment elevation myocardial infarction (STEMI). The aim of this study was to identify possible predictors for no-reflow.

**Patients and methods:**

We investigated 218 patients with acute anterior STEMI who underwent PPCI from December 2016 to December 2018. No-reflow was defined as a coronary TIMI flow grade of ≤ 2. TIMI flow grade 3 was defined as normal reflow.

**Results:**

In our study, the no-reflow phenomenon was observed in 39 patients (18%) during angiography. The patients of no-reflow group were found to be more older, diabetics, longer pain-to-balloon time, lower blood pressure, higher platelet counts and higher levels of D-Dimer and Cystatin C (Cys-C). In multivariate logistic regression analysis, only diabetes (OR = 0.371, 95% CI: 0.157–0.872, P = 0.023), longer pain-to-balloon time (OR = 1.147, 95% CI: 1.015–1.297, P = 0.028) and higher Cys-C level (OR = 10.07, 95% CI: 2.340–43.377, P = 0.002) were predictors for no-reflow.

**Conclusion:**

Cys-C might be a useful predictor for the no-reflow phenomenon after PPCI in STEMI patients. It might help to screen STEMI patients with high risk of no-reflow on admission.

## Introduction

The best treatment of ST-segment elevation myocardial infarction (STEMI) is reperfusion of ischemic myocardium as soon as possible. Primary percutaneous coronary intervention (PPCI) has become the preferred strategy for reperfusion and the current standard care for STEMI[1]. Nevertheless, about 12% to 32.8% [2] of STEMI patients performed with PPCI do not achieve desired coronary blood flow, which is referred to as the no-reflow phenomenon[3]. No-reflow is clinically important as it is associated with cardiac failure, malignant arrhythmias and in-hospital and long-term mortality. Multifactorial factors may contribute to the development of no-reflow including distal embolization, vasospasm, microvascular damage, oxidative stress, and ischemia-reperfusion injury[4–6]. But the predisposing factors for the no-reflow phenomenon are still not thoroughly understood.

Cystatin C (Cys-C) is the most important inhibitor of endogenous cysteine proteases and serves as a marker of renal function[7]. Epidemiological studies show that Cys-C is associated with cardiovascular diseases, such as atherosclerosis, heart failure, ischemic stroke and acute coronary syndrome[8–13]. High Cys-C level is indicated as a useful marker for identifying an elevated risk of cardiovascular diseases, and is independent of renal function determined by creatinine. In the present study, we also investigated the relationship between Cys-C and no-reflow in patients with STEMI who are undergoing PPCI.

## Subjects and methods

### Ethics statement

The protocol of the present study was approved by the Ethical Committees of the Second Hospital of Shandong University (approval number KYLL-2016A-0041) and the study conformed to the Declaration of Helsinki Principles. Informed written consent was obtained from all participants. The survey was completely anonymous and did not ask for identifying information.

### Study population

The study population was composed of 218 patients with acute anterior STEMI who underwent PPCI in the Second Hospital of Shandong University from December 2016 to December 2018. Venous blood samples were obtained at admission. All patients met standard diagnostic criteria and received PPCI of the left anterior descending artery (LAD) within 12 h from symptoms onset. The key exclusion criteria were as follows: age >75 years; PPCI was performed after 12 h from symptoms onset or no stent was implanted during the PCI; thrombolysis failure and rescue PCI; cardiogenic shock; acute pulmonary edema; ventricular septal rupture; cardiac tamponade; severe respiratory, renal, or hepatic dysfunction or failure; history of thromboembolic disease and imflammatory process.

### Angiographic analysis and PPCI

All patients received 300 mg acetylsalicylic acid (ASA), 600 mg clopidogrel and 40mg atorvastatin as a loading dose on admission and intravenous standard heparin (70 U/kg of body weight) before CAG/PCI. The procedure was performed with standard technique and the radial artery approach was the first choice. The glycoprotein IIb/IIIa receptor inhibitor tirofiban was administered during the PPCI according to the operator’s preference. Balloon predilatation or postdilatation, the type of stents, and thrombus aspiration were used according to the operator’s discretion. The blood flow in the infarct-related artery (IRA) was measured following stenting during the angiography according to the Thrombolysis in Myocardial Infarction (TIMI) grading system[14]. No-reflow was defined as a coronary TIMI flow grade of ≤ 2. TIMI flow grade 3 was defined as normal reflow.

### Laboratory analysis

Blood sample was drawn from the antecubital vein in the emergency department. A routine laboratory blood work-up was conducted for all of the patients.

### Statistical analysis

All the descriptive variables are expressed as the mean ± standard deviation (SD). Categorical data are expressed as frequencies and percentages and the Chisquare test or Fisher’s exact test was used for the analysis. Comparisons between 2 groups were conducted using the Student’s *t* test when the variables were normally distributed, and Mann–Whitney U test was used for abnormal distribution. Multivariable logistic regression analysis was applied to identify independent predictors of no-reflow. Variables that could be a predictor of no-reflow with a significant p value were entered into multivariate analysis. The results of univariate and multivariate regression analyses were presented as odds ratio with 95% CI. A two-tailed p value of < 0.05 was considered statistically significant. The above statistical analyses were performed using SPSS 19.0 (IBM, Chicago, IL, USA).

## Results

### Baseline clinical characteristics of patients

PPCI was performed in 218 STEMI patients during 2016–2018. The 218 patients were divided into two groups according to the final TIMI flow after the PPCI. No-reflow was seen in 39 (18%) patients during angiography. The clinical characteristics of the subjects in two groups were shown in Table 1. There was no statistically significant difference between normal reflow group and no-reflow group in gender, BMI, active smokers, hypertension and PCI history. Patients with no-reflow were older as compared to normal reflow group (63.41±9.12 years vs. 57.73±10.65 years, p = 0.002). Proportion of elderly patients over 70 years is higher in no-reflow group (11.17% vs. 25.64%, p = 0.017). The prevalence of diabetes was significantly higher in the no-reflow group than in the normal reflow group (25.14% vs. 48.72%, p = 0.003). Pain-to-balloon time is significantly longer in no-reflow group (5.28±2.99 hours vs. 7.15±3.83 hours, p = 0.006). No-reflow patients had lower blood pressure at admission (128.83±21.64 vs. 120.38±17.35 mmHg, p = 0.024) and likely more history of hypertension (53.85% vs. 50.84%).

**Table 1:** Baseline demographic and clinical parameters of two groups.

	Normal-reflow (n = 179)	No-reflow (n = 39)	*p* value
Age (years)	57.73±10.65	63.41±9.12	**0.002**
Age > 70 y, n(%)	20 (11.17)	10 (25.64)	**0.017**
Male, n (%)	150 (83.80)	30 (76.92)	0.305
BMI (kg/m^2^)	25.43±1.28	25.42±1.14	0.970
Current smoker, n (%)	109 (60.89)	26 (66.67)	0.501
Hypertension, n (%)	91 (50.84)	21 (53.85)	0.733
Diabetes, n (%)	45 (25.14)	19 (48.72)	**0.003**
History of PCI, n (%)	9 (5.03)	1 (2.56)	0.807
Pain-to-balloon time (hour)	5.28±2.99	7.15±3.83	**0.006**
SBP (mmHg)	128.83±21.64	120.38±17.35	**0.024**
Heart rate (beats/min)	77.58±17.38	76.79±17.94	0.799
LVEF (%)	52.63±5.89	51.44±4.71	0.799

Values are given as mean ± SD or n (%). BMI, body mass index; PCI, percutaneous coronary intervention; SBP, Systolic blood pressure; LVEF, Left ventricular ejection fraction.

### Laboratory characteristics of patients

As presented in Table 2, no significant differences between patients in no-reflow group and normal reflow group were detected in WBC counts, hemoglobin, blood lipids (TG, TC, LDL-C, HDL-C), glucose, eGFR and hs-CRP. Higher values of platelet counts were detected in the no-reflow group (p = 0.032). The levels of D-Dimer were higher in no-reflow group than the normal reflow group (p = 0.015). The serum levels of Cys-C in patients with no-reflow was significantly higher compared to the normal reflow group (0.89±0.21 mg/L vs. 1.10±0.38 mg/L, p = 0.001).

**Table 2:** Comparison of laboratory data in two groups.

	Normal-reflow (n = 179)	No-reflow (n = 39)	*p*value
WBC (10^9^/L)	10.57±3.29	10.29±2.80	0.613
HGB (g/L)	139.46±20.96	136.72±25.77	0.478
PLT (10^9^/L)	245.96±66.00	270.64±57.74	**0.032**
TG (mmol/L)	1.72±1.11	1.73±0.99	0.981
TC (mmol/L)	4.65±0.94	4.63±0.87	0.900
LDL-L (mmol/L)	2.99±0.73	2.81±0.72	0.156
HDL-L (mmol/L)	1.07±0.26	1.12±0.23	0.318
FBS (mmol/L)	8.23±5.01	7.63±3.19	0.476
eGFR (ml/min /1.73 m^2^)	104.70±24.47	100.33±21.18	0.303
Cys-C (mg/L)	0.89±0.21	1.10±0.38	**0.001**
hs-CRP(ng/ml)	14.20±10.60	15.02±10.35	0.663
D-Dimer (ng/ml)	410.35±237.28	536.87±291.69	**0.015**

Values are given as mean ± SD. WBC, white blood cells; HGB, hemoglobin; PLT, platelet; TG, triglycerides; TC, total cholesterol; LDL-C, low-density lipoprotein cholesterol; HDL-C, high-density lipoprotein cholesterol; FBS, fasting blood sugar; eGFR, estimated glomerular filtration rate; Cys-C, cystatin C; hs-CRP, high sensitivity C-reaction protein.

### Univariate and multivariate logistic regression

The effects of different variables on no-reflow were analyzed by using univariate and multivariate logistic regression analyses as shown in Table 3. In univariate analysis, elder age (OR = 1.056, 95% CI: 1.019–1.095, P = 0.003), diabetes (OR = 0.343, 95% CI: 0.168–0.701, P = 0.003), longer pain-to-balloon time (OR = 1.174, 95% CI: 1.060–1.301, P = 0.002), lower blood pressure (OR = 0.979, 95% CI: 0.960–0.997, P = 0.026), higher platelet counts (OR = 1.006, 95% CI: 1.000–1.011, P = 0.034), higher D-Dimer level (OR = 1.002, 95% CI:1.000–1.003, P = 0.007) and higher Cys-C level (OR = 16.849, 95% CI: 4.481–63.357, P<0.001) were predictors for no-reflow. Next we established a multivariable logistic regression model by using no-reflow as the dependent variable with adjustments for significant variables. In multivariate logistic regression analysis, only diabetes (OR = 0.371, 95% CI: 0.157–0.872, P = 0.023), longer pain-to-balloon time (OR = 1.147, 95% CI: 1.015–1.297, P = 0.028) and higher Cys-C level (OR = 10.07, 95% CI: 2.340–43.377, P = 0.002) were predictors for no-reflow.

**Table 3:** Univariate and multivariate logistic analysis for no-reflow.

	Univariate			multivariate		
	OR	95% CI	*p* value	OR	95% CI	*p* value
Age	1.056	1.019–1.095	**0.003**	1.037	0.981–1.096	0.196
Age > 70	0.365	0.155–0.859	**0.021**	1.236	0.315–4.842	0.761
Male	1.552	0.667–3.610	0.308			
BMI	0.995	0.753–1.314	0.970			
Current smoker	0.645	0.307–1.356	0.248			
Hypertension	0.886	0.443–1.775	0.734			
Diabetes	0.343	0.168–0.701	**0.003**	0.371	0.157–0.872	**0.023**
History of PCI	2.012	0.247–16.358	0.513			
Pain-to-balloon	1.174	1.060–1.301	**0.002**	1.147	1.015–1.297	**0.028**
SBP	0.979	0.960–0.997	**0.026**	0.981	0.961–1.002	0.077
Heart rate	0.997	0.978–1.017	0.798			
LVEF	0.966	0.913–1.023	0.241			
WBC	0.972	0.871–1.085	0.611			
HGB	0.995	0.981–1.009	0.479			
PLT	1.006	1.000–1.011	**0.034**	1.006	1.000–1.013	0.060
TG	1.004	0.730–1.380	0.981			
TC	0.976	0.671–1.419	0.900			
LDL-L	0.703	0.431–1.145	0.157			
HDL-L	1.972	0.521–7.466	0.317			
FBS	0.962	0.866–1.069	0.474			
eGFR	0.992	0.978–1.007	0.302			
Cys-C	16.849	4.481–63.357	**<0.001**	10.07	2.340–43.377	**0.002**
hs-CRP	1.007	0.976–1.039	0.662			
D-Dimer	1.002	1.000–1.003	**0.007**	1.001	1.000–1.003	0.053

### The ROC curve of Cys-C for predicting no-reflow

**Fig 1:**
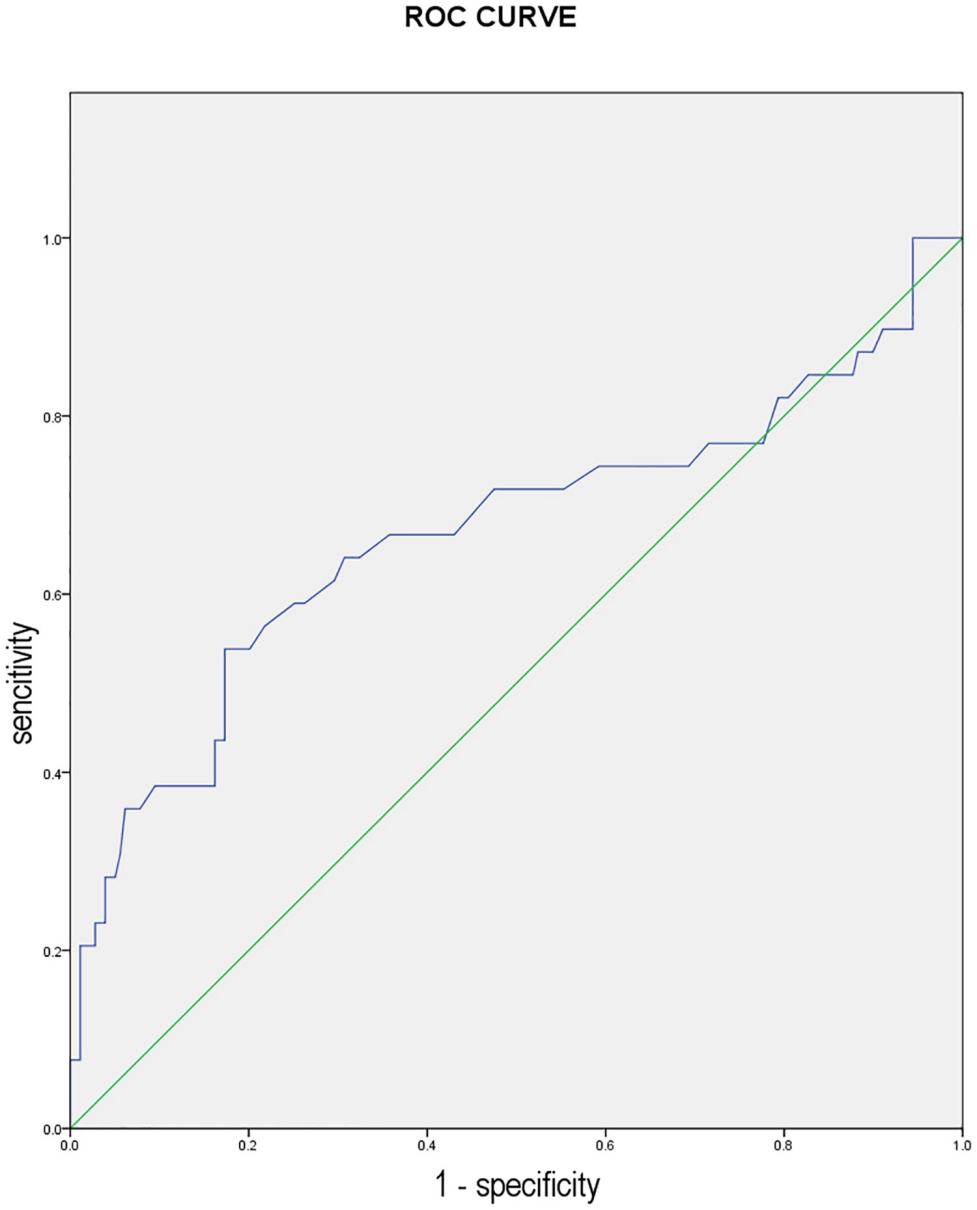
The receiver operator characteristic (ROC) curve analysis of cystatin C in predicting no-reflow phenomenon. (AUC, 0.668; 95% CI, 0.557–0.780; *p* = 0.001).

To further evaluate the value of using Cys-C as a predictive marker for the no-reflow phenomenon after PCI, we performed the ROC analysis. As shown in Fig 1, a Cys-C level of > 1.055 mg/L, had 54% sensitivity and 83% specificity in predicting the no-reflow event. The AUC of Cys-C was 0.688 with a 95%CI of 0.557–0.780.

## Discussion

We have demonstrated in the present study that Cys-C is associated with no-reflow phenomenon in patients with PPCI. Our results suggest that serum Cys-C level may be a new predictor of no-reflow in STEMI patients treated by PPCI.

Early revascularization of infarct-related artery by PPCI has become the most effective strategy in STEMI patients. But no-reflow phenomenon significantly limits the benefits of PPCI therapy. Detecting clinical predictors for insufficient myocardial reperfusion may help STEMI patients with no-reflow. In our study, the rate of no-reflow after PPCI is about 18%, and it is similar to the rates of no-reflow reported in previous studies varied from 2.3 to 39.9% [15–18]. Patients with no-reflow have an increased incidence of ventricular early congestive cardiac failure, arrhythmias and cardiac death. No-reflow has been shown to be associated with worse short-term and long-term mortality[19, 20]. Currently, there is still lack of targeted therapy to reverse the no-reflow phenomenon. Therefore, it is important to predict and prevent the no-reflow phenomenon with PPCI. Several recent studies have shown that some clinical biomarkers and parameters can predict the risk of no-reflow phenomenon, such as the WBC count[21], D-dimer[22], neutrophil/lymphocyte ratio[23] and CHA2DS2-VASc score[24]. In the present study, the patients of no-reflow group were found to be more older, diabetics, longer pain-to-balloon time, lower blood pressure, higher platelet counts and higher levels of D-Dimer and Cys-C. But with multivariate logistic regression analysis, only diabetes, longer pain-to-balloon time and higher Cys-C level were predictors for no-reflow. Diabetes increases the risk of complications in STEMI patients through endothelial dysfunction and platelet dysfunction[25, 26]. Some studies suggested that diabetes reduced coronary collateral development[27]. Delayed pain-to-balloon time is associated with greater ischemia/reperfusion injury which leads to oxidative stress, inflammatory response, oedema of capillary bed and myocardial cells swelling[28]. Previous studies have shown that pain-to-balloon time ≥ 6h was independently associated with no-reflow[16, 29]. Lower pain-to-balloon time may be a potentially preventable factor for no-reflow. Further, Cys-C level was found to be associated with no-reflow in the present study.

Recent evidence suggests that high circulating Cys-C is associated with cardiovascular diseases, independent of creatinine-based renal function estimates. In addition, heritability analyses indicate that Cys-C and cardiovascular diseases share genetic influences[30]. Cys-C is a cysteine protease inhibitor produced by most human cells and it is a sensitive measure of renal function that be less affected by sex, age and lean muscle mass than creatinine[31, 32]. Previous studies showed that a higher Cys-C level was associated with unfavourable cardiovascular outcomes in STEMI patients treated with PPCI, including impaired myocardial perfusion, poor cardial functional recovery, cardiogenic shock and death[33, 34]. The present study demonstrated that admission Cys-C levels are independently associated with no-refow in patients with STEMI treated with PPCI. Several underlying mechanisms may be involved in the probability that Cys-C predict no-refow after PPCI. First, as we all know, high Cys-C levels suggest mild to severe renal dysfunction. Mild renal dysfunction is associated with microvascular endothelial dysfunction, oxidative stress, procoagulant cytokines and free radical. And all these factors participate the development of poor myocardial perfusion after PPCI[17, 35]. Second, high Cys-C levels may contribute to no-refow by regulating inflammation. It has been suggested that high Cys-C concentrations are directly related to inflammation[36, 37]. Infammation has been well recognized to play an important role in the development of no-refow.

Based on our results, we conclude that Cys-C might be a useful predictor for the no-reflow phenomenon after PPCI in STEMI patients. It might help to screen STEMI patients with high risk of no-reflow on admission and help to choose the best treatment.

## Study limitations

Several limitations should be mentioned for the present study. First is its modest sample size, further studies in more patients are needed to confirm our data. Second, for all the enrolled patients, LAD was the culprit vessel. Our results might be confirmed with other coronary arteries. Third, the association and changes in Cys-C over time were not studied. The prognostic impact of Cys-C in no-reflow of STEMI patients remains to be examined in future studies.

## Supporting information

S1 Data Set Full data set. (SAV)
